# Primary care occupational, physical, and respiratory therapy role adaptation in the first year of the COVID-19 pandemic

**DOI:** 10.1186/s12875-023-02247-7

**Published:** 2024-01-02

**Authors:** Cara L. Brown, Louise Chartrand, Brontë Vollebregt, Dayajyot Kaur, Tory Crawford, Patricia Thille

**Affiliations:** 1 Department of Occupational Therapy, College of Rehabilitation Sciences, Rady Faculty of Health Sciences, University of Manitoba, R125, 771 McDermot Ave, Winnipeg, Manitoba R3E 0T6 Canada; 2 Department of Respiratory Therapy, College of Rehabilitation Sciences, Rady Faculty of Health Sciences, University of Manitoba, Winnipeg, Manitoba Canada; 3 Applied Health Sciences Program, Faculty of Graduate Studies, University of Manitoba, Winnipeg, Manitoba Canada; 4 Rehabilitation Sciences Program, College of Rehabilitation Sciences, Rady Faculty of Health Sciences, University of Manitoba, Winnipeg, Manitoba Canada; 5 Department of Physical Therapy, College of Rehabilitation Sciences, Rady Faculty of Health Sciences, University of Manitoba, Winnipeg, Manitoba Canada

**Keywords:** Occupational therapy, Physical therapy, Respiratory therapy, Primary care, Rehabilitation, Telehealth, Role adaptation, COVID-19 pandemic, Role clarity, Disaster preparedness

## Abstract

**Background:**

Occupational, physical and respiratory therapists are relatively new to primary care settings, and thus their roles are still emerging. The COVID-19 pandemic was a time of abrupt changes in professional roles. Professional role adaptations are integral to the ability of health care teams to respond to day-to-day care delivery challenges, such as the current physician and nurse shortage, as well as disaster situations. This study explored the role adaptation of occupational, physical, and respiratory therapists in Canadian primary care settings throughout the first year of the COVID-19 pandemic, as well as barriers and facilitators to adaptation.

**Methods:**

This longitudinal interpretative descriptive study purposively sampled primary care occupational, physical, and respiratory therapists from two Canadian provinces (Manitoba and Ontario). We asked participants to prepare at least 10 semi-structured audio-diary entries during a 12-week period (April – Oct 2020), followed by two semi-structured interviews (Dec 2020, Apr 2021). Questions focused on changes happening in their practice over time. Analysis was iterative, including developing a individual summaries and coding data using both inductive and pre-determined codes. We then entered an immersion/crystallization process to develop key themes related to role adaptation.

**Results:**

We represent our findings with the metaphor of the game of *Role Adaptation Snakes and Ladders (*aka *Chutes and Ladders)*. The pandemic was certainly not a game, but this metaphor represents the tension of being a pawn to circumstance while also being expected to take control of one’s professional and personal life during a disaster. The object of the game is to move through three phases of role adaptation, from *Disorienting*, through *Coping and Waiting*, to *Adapting*. In the *Adapting* phase, the therapists creatively found ways to provide vital services for the pandemic response. The therapists were influenced both negatively and positively (snakes and ladders) by their personal circumstances, and professional meso and macro contexts. Each therapist moved across the board in a unique trajectory and timeline based on these contexts.

**Conclusions:**

Rehabilitation professionals, with adequate meso and macro system supports, can maximize their role on primary care teams by adapting their services to work to their full scope of practice.

**Supplementary Information:**

The online version contains supplementary material available at 10.1186/s12875-023-02247-7.

## Background

In the last two decades, occupational therapists, physical therapists, and respiratory therapists have joined primary care teams in some Canadian provinces. Their presence enhances primary care’s comprehensiveness and responsiveness to community needs, both key aims of primary care reform in Canada [1]. Like family physicians and nurse practitioners, occupational physical, and respiratory therapists (hereafter referred to collectively as “primary care therapists”) work across the lifespan, offering care for acute and chronic physical and mental health issues [2–5]. In addition, they can provide specialized rehabilitative assessment and treatment for respiratory, cardiovascular, neurological, musculoskeletal, and psychological concerns to promote and restore health and functional abilities [4, 6–8]. This is especially valuable given limited access to other publicly funded community-based occupational, physical and respiratory therapy in Canada. Research has primarily focused on describing the current roles of primary care therapists [4, 9] However, primary care physicians’ understanding of therapists’ roles is quite narrow [5, 7, 10] in comparison to their full scope of practice. Since primary care therapy is primarily physician referral driven, this research does not necessarily provide us with knowledge on the extent to which primary care therapists can support team-based primary care. To realize the benefits of team-based primary care, primary care systems need to maximize all team member roles to best meet population needs [11].

With usual clinical routines disrupted during the pandemic, clinicians and teams needed to adapt care delivery to meet public health orders and new population needs. Thus, the COVID-19 pandemic provided an opportunity to examine how adaptation occurs, including role adaptation, to fit the new circumstances. Most of the literature on role change during the pandemic focuses on the transition to virtual care [12, 13], addressing the mode of service delivery more than the type of service delivered. Some studies describe the changes in primary care physician roles during the COVID-19 pandemic, including screening, triage, patient education, and doing more specialty care [14, 15]. There is little that focuses specifically on primary care therapists’ role change during the COVID-19 pandemic. The latter is important to understand for both future disaster response and to realize the potential of team-based primary care.

Therapists are well positioned to aid in disaster preparedness, response, and recovery, but their contributions are less recognized and researched. There is less understanding of their roles and responsibilities [16], they are missing in disaster response plans [17], and the understanding of their contributions is limited to addressing musculoskeletal injuries and mental health. Therefore, primary care therapists had little guidance for role adaptation during the COVID-19 pandemic, a setting in which usual roles of therapists are not yet clearly defined. While addressing musculoskeletal injuries and mental health care are important, this limited understanding of primary care therapist potential roles creates underutilization of these professions to meet population health needs [18–21].

Beyond disasters, the nursing and physician shortage in Canada intensifies the need to maximize the use of other health professionals in primary care [22]. In both day-to-day primary care delivery, and in response to an event like a disaster, the resources within the clinic shape what services are possible to provide. Primary care physicians had difficulty keeping up with meeting the primary care needs of their patients [14, 15], and unfortunately, the pandemic heightened the physician shortage, with primary care doctors leaving their practice during the pandemic due to burnout and retirement [22]. Team-based care can help by distributing tasks among the team to maximize their impact on health promotion and prevention as well as disease management [23]. For good team function, it is important for each team member to understand the breadth of scopes of their respective professions, and how these roles can adapt to different needs of the team, the population being served, and the context [3, 7, 22, 24, 25]. There is much learning to be done about the roles of therapists in primary care, and how to support role adaptation to support the goals of the primary care team.

We undertook this study to explore the role adaptation that occurred in Canadian primary care settings for occupational, physical, and respiratory therapists during the COVID-19 pandemic, and barriers and facilitators to this adaptation. Doing so advances knowledge in the contributions rehabilitation professionals can make in primary care settings and/or during disasters, providing insight into how their roles can be optimized in day-to-day care. Furthermore, it provides insight into how to support the role adaptation of therapists so that they can move more quickly from a state of disorientation to a state of adaptation during change.

## Methods

### Methodology and design

We used an interpretative description methodology to study role adaptation of primary care therapists during the first year of the COVID-19 pandemic. Consistent with constructivism, interpretive description appreciates the context-specific aspects of how people make meaning of their experiences [26]. Thus, interpretive description anticipates the possibility of both commonalities and variation, given context variation [26]. The methodology can integrate existing theory but need not do so. Interpretive descriptive studies aim to create meaningful and practical results that are applicable to real world settings in which they are relevant [26, 27]. We used audio-diaries and two interviews to collect longitudinal data (described further below in Data Collection section).

### Theoretical influences

Our understanding of role adaptation is informed by Turner’s role theory [28]. Turner theorizes roles as a combination of attitudes and behaviors that are guided by implicit or explicit expectations at an individual or group level [28]. There are various types of roles, including basic roles (such as age or gender), position or status roles (such as family roles and occupations) and functional group roles (such as a leader, follower, or counsellor) [28]. Roles within professional organizations, such as in health care, form a framework to organize work responsibilities [28]. Roles encompass a relational quality in which one role relates to another, such as patient/clinician, or roles within teams that can evolve over time.

The COVID-19 pandemic was a time of abrupt changes in professional and personal roles. Abrupt role changes tend to elicit feelings of unease for individuals impacted by the change [28]. Role adaptation involves a change of self, of behaviors and attitudes, and externally, of performing different tasks and re-orienting relationships with others [28]. Studies exploring other situations requiring role adaptation have found that this transition can be uncomfortable and lead to negative effects on an individual’s professional performance and personal wellbeing [29]. Further, health professionals can have a loss of self-identity and confidence when working in different environments and being assigned new responsibilities [30]. This negative aspect of role adaptation can involve feelings of anxiety, fear of failure, and burnout, which can lead to a decreased level of confidence in the adaptation of existing roles and the transition to new roles [29]. Yet professional role adaptations are integral to the ability of the health team to respond to fluctuations in population needs, and can improve the quality of care, for example, increasing the team’s ability to provide holistic care [30]. Therefore, understanding what best supports individuals during role adaptation is important to be able to support individuals who are undergoing, or are being asked to undergo role adaptation.

Duchscher [29] studied the professional role adaptation of nurses transitioning into practice. While they focused on a different type of professional role adaptation than us,  they theorize contextualizing factors that contribute to role adaptation, including numerous personal and environmental factors that prescribe and cultivate expectations about professional roles and responsibilities that are potentially applicable to multiple types of professional role adaptation. These factors include knowledge of one’s role in particular contexts, one’s ability to develop and sustain collaborative relationships, organizational structure, and peer support [29].

One way to look at social systems and the relationships and interactions within them is to use a micro-meso-macro framework [31]. This can provide a useful lens for understanding influences on, and between, different social organizational levels. In this study, we situate the individual therapist – as an employee, health services provider, and family member and friend - at the micro level. We interpret the meso level as the immediate organizational aspects of primary care such as team communication, relationships within the local organization, and local work policies and procedures. At the macro level, we place the external and highest levels of the health system governance, such as the provincial government or related administrative organization for the health care system.

### Setting and recruitment

This study explored the experiences of occupational, physical, and respiratory therapists during the first year of the COVID-19 pandemic in two Canadian provinces: Manitoba and Ontario. Canada has a universal health care system in which the most common family physician model is fee-for-service, where the government reimburses family physicians for patient care [32]. Publicly funded interprofessional primary care services are a more recent addition to public services. They are not consistently accessible to the Canadian population. In Ontario, team-based primary care is delivered in several different models including family health teams (FHTs), first implemented in 2005, that now serve 25% of Ontario’s population, and community health centres (CHCs) [33], among others. Interprofessional primary care teams are newer in Manitoba, with My Health Teams first implemented in 2014 [34]. At the time of this study, there were 13 My Health teams serving four of the five health regions in the province of Manitoba. Each of those health regional teams were granted responsibility to operationalize the primary care reform the way they saw fit, to serve their respective community. There were a handful of other therapists in primary care in settings other than My Health Teams, such as family residency teaching clinics and Access centres (health and social care hubs).

Given the time-sensitive nature of recruitment at the start of the COVID-19 pandemic, we used multiple methods to find participants that fit our purposive criteria. We sought to enrol two to four participants by each profession and province, to ensure an adequate sample to support comparison across provinces and professions. In Manitoba, we circulated the recruitment notice directly to primary care therapists we knew (almost the complete population) and asked that they share with others. In Ontario, two research colleagues who are well connected with primary care allied health shared the recruitment notice on our behalf. As recruitment continued, we identified a gap of physiotherapists in Ontario, so created a list of primary care team-based physiotherapists and emailed the study notice to them directly. When a person contacted the research team, one of two investigators met with them via Zoom to share info about the project, and if they were still interested, to orient them to starting diary entries.

### Data collection and early analysis steps

We collected qualitative data longitudinally, using two methods in sequence: audio-diaries and interviews. Consistent with constructivist research, diary-interview studies provide insight into how participants make sense of events in an ongoing way [35]. Data collection and analysis were iterative, so that the emerging insights from diaries could be further explored in the interviews over the course of the year. We collected the following demographics: profession, province in which they practice, and duration of time practicing in primary care settings.

Diaries allow participants to share their experiences and interpretations of real-life events close to their actual occurrence, as a ‘view from within’ [35]. Starting in late April 2020, we asked participants to audio-record a diary once or twice a week, for a minimum of 10 diaries over 12 weeks, a time-based approach to diary writing [36]. For the first entry, we asked participants to summarize the time passed since the start of the pandemic, after which they shifted to recording entries focused on the current day. We created a semi-structured diary guide (Appendix A), with four open-ended questions to explore new ways of working, and impacts on them, their work relationships, and overall work environment from the COVID-19 pandemic. We added a fifth question partway through, asking explicitly about how the changing work environment was impacting work relationships and teamwork, a topic some were spontaneously speaking about.

After transcription of audio diaries was complete for a participant, a research team member created an individual summary of their case, using structured headings:

1. What are the biggest practice changes that occurred for this participant from the start of the pandemic, through to the end of the diary entries?
2. Which of these practice changes did they initiate themselves?
3. What are their biggest challenges they’re facing?
4. Other notes (distinguishing characteristics or events).

The investigators then read the full set of individual case summaries to generate ideas about topics to explore in interviews beyond those immediately relating to our objectives. We – the investigators and two masters students– then drafted an interview guide, integrating both research objective related topics and emergent lines of inquiry. The objective of the follow-up interviews was to invite broader reflection on the everyday events described in the audio diaries, as well as invite a description of the present [37].

We piloted the first interview guide on two occasions. Five team members then interviewed participants using video-meeting platforms in December 2020/January 2021 – the investigators and two masters students, with direct supervision of the first interviews led by the students. After the interview, a team member updated the individual case summary. We repeated these steps to prepare for the second interview: immersive reads of individual cases, updating the interview guide to further integrate emerging findings, and piloting/training student interviewers prior to initiating interviews in April 2021, then transcription and review of transcripts for accuracy. For more information on the methods, please see Thille et al. [38].

### Data analysis

We used multiple strategies to organize and interpret data to support analysis over time, in an approach called Immersion-Crystallization [39]. In addition to the individual case summaries developed from immersive reads of the data, we each wrote analytic memos and had regular team analysis meetings. We drafted codes that addressed both the research objectives and early analytic insights we wished to explore, refining after piloting. After first-level coding completion of diaries and interviews, the investigators read the data contained within the respective codes, writing a summary to highlight commonalities and variations within, and deciding what topics needed to be coded within the larger node. Through cycles of coding, reviewing, and analytic discussions, we co-interpreted the data about role adaptation.

### Ethics

This study was approved by the institutional HREB H2020:270 (HS23987). Of note, the board approved recruitment of participants to start audio-diaries prior to receiving the final ethics approval certificate given the time sensitive nature of this data, with the proviso that the participants could not submit diaries to the research team until ethical approval and written consent was obtained.

## Findings

**Fig. 1:**
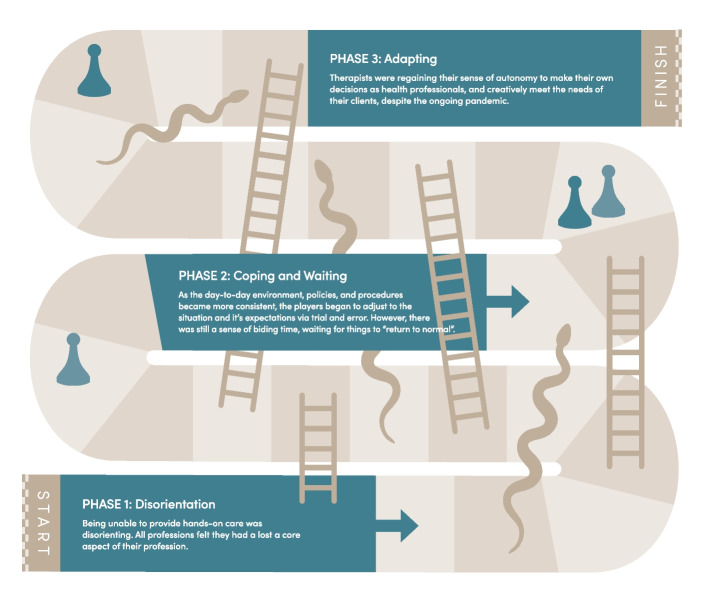
Role Adaptation Snakes and Ladders. Image credit: Jess Koroscil

We present our findings by using the metaphor of a game of *Role Adaptation Snakes and Ladders* (aka Chutes and Ladders - See Fig. 1). While the pandemic was certainly not a game, this metaphor represents the tension of being a pawn to circumstance while also being expected to take control of one’s daily work and personal life in the middle of a disaster. The therapists are the metaphorical *players* in the game. They were influenced by the various systems and relationships in which they are embedded, and role adaptation was shaped over time by changes in the pandemic and policies. We chose this *Snakes and Ladders* metaphor to reflect patterns in our data, both in the therapists’ respective accounts over the course of the year, and patterns in external influences that impacted role adaptation.

The object of the game of *Role Adaptation Snakes and Ladders* is to move through three phases of role adaptation, starting in the first phase, *Disorienting*, moving upwards on the board through *Coping and Waiting*, aiming to reach the final phase, *Adapting*. The therapists’ trajectory is influenced by their personal and professional circumstance, represented by the numbers rolled with the dice, and meso and macro influences, which are represented by the snakes and ladders. Each therapist thus moves across the board (through the phases of role adaptation) in their unique trajectory and timeline based on their individual and meso-macro contexts.

Despite their individual differences, we found that all therapists moved through the first two phases, with some reaching the third phase, even if in only some areas of their practice. Despite their unique role adaptation trajectories, the therapists had similar meso and macro influences which we represent by the snakes and ladders. We will now describe each of the game phases and elements in detail to further explain our findings.

### The therapists

Sixteen therapists participated in the study, nine from Ontario and seven from Manitoba. There were five occupational therapists, six physical therapists, and five respiratory therapists. For each profession, three of the therapists had worked in a primary care setting for more than 2 years. Three physical therapists had less than 3 months on their team when the pandemic started. All 16 completed audio diaries, 13 completed the first interview, and 10 completed the second interview. We did not collect reasons for attrition from those who did not complete all three components of the study, but some reasons provided were no longer practicing in primary care, and ongoing redeployment outside of primary care. The second interview was not in the original research plan, so attrition was not unexpected at this stage.

### Phase 1: Experiencing disorientation in a previously familiar role

All therapists entered the game at the disorienting phase. Within a week or two, they were told they needed to stop providing in-person care and were asked to start working from home. Tara describes this time:

My work did change significantly in that second half of March. I think the change was quite dramatic. The biggest change for me was not providing care to clients or having to cancel appointments. I think, of course, that was the most safe and right thing to do, but I think discontinuing care always has a bit of a… It doesn’t feel normal. It’s not something I’ve ever had to do before where I’ve had to cancel a patient’s appointment just out of the blue, and then not being able to offer an alternative very quickly that might be satisfactory. So, I think that was something that just made me feel not very professional, but I know that it was the safe thing to do as well. Yeah, I was making a lot more phone calls as well to clients, and emails, which is something different. I usually don’t do that in my practice but needing to do that in that second half of March was quite significant. Working from home was something I would never have imagined doing as a physiotherapist, but that started to take into gear in the last week of March.

The therapists immediately had fewer tasks to do which impacted their overall sense of their role. Emma’s speaks to the impact of change of her tasks on her role identity:

Yeah, so basically, all the things that people (a) knew me for… it’s just like all of my usual stuff has changed. Then I’m also working from home now. Sooooo, yeah, basically, everything feels like it’s flipped. Again, I might be a little dramatic today because I’m not really in my best mood, but, yeah, just feel like everything’s flipped.

Early in the pandemic, each therapist described losing the ability to provide an essential and core component of their profession. From a wider health system perspective, many of the services provided by therapists in primary care were not considered essential or urgent, and thus these professions were asked to refrain from providing in-person care because of pandemic policies. This left the primary care therapists feeling unmoored as they could not carry out activities central to their role. Occupational therapists lost the ability to assess people’s function and participation in their home and community environments; assessing people in context is a key feature of their profession. This tension is seen in this comment from Emma, who is trying to reduce her home visits as another COVID-19 wave starts but is finding it difficult.

I’ve been thinking more about maybe [sigh] trynna see people less in their homes again. Um, I think I've been- it's just, I find it’s very hard to, uh, meet with people by phone and I don't really enjoy it very much and so, um, I've been doing a little bit but when it's more sort of home visit stuff, I often just do the majority of the home visit in the person's home, when I probably should be doing more by phone. Um, so, yeah, just kind of being more thoughtful about that. Um, it’s sort of a hard balance ‘cause I feel like I'm wearing the PPE (personal protective equipment), I'm, you know, I'm available in there, so I might as well do my sort of thorough assessment, um, ‘cause often in my assessment, I get people to, kind of, move and do things while I'm getting information.

Physical therapists lost their core activity of providing hands-on physical assessment and care. They quickly started using the telephone and/or providing virtual visits to conduct consultations or follow-up with people already on their caseload. However, the physical therapists saw this as a less-than-ideal care provision model and as a temporary measure. The frustration with not being able to support in-person physical care is evident in Tina’s comment, as she thinks back to the first month of the pandemic:

You know, especially in the beginning, it was frustrating, because I was thinking to myself that -Oh I could easily just do this um, manual therapy in-person. Or I could easily show them an exercise in-person, and then have them do it, or observe how they were moving- And like, all of those things are really important that I would use for myself. And because I can’t see them, if I did over the phone, or virtually, it is harder. Because you can’t see all the small minor details. Um, it was… it was very um, frustrating and very difficult.

Respiratory therapists lost their primary role of conducting in-person assessments, the largest loss being spirometry assessments – a key feature of their profession to support and orient respiratory health care. When Heidi reflected on her work early in the pandemic and how it compared to pre-pandemic work, she said:

Not similar at all. I’d never done a phone or virtual appointment ever, and once the pandemic hit, that was all I was supposed to do. Almost every patient I saw would have a spirometry done pre-pandemic, and as soon as I stopped testing on March 26th, I couldn’t do anymore spirometry. The only thing I continued to try and do was education, but the education, itself, changed, because it became over the phone. A lot of the education I try to do would be very hands-on and it would be inhaler technique, so you’d be showing a patient a device and how to use it, and now I was having to explain over the phone. We didn’t even have Zoom set up, and we didn’t even have the ability to see people. Even the education, itself, although I was trying to educate on similar things, I had to change how I did the education, because I could no longer use my tools to show patients what to do. It completely changed. It wasn’t similar at all.

As time passed, spirometry waiting lists grew longer, leaving respiratory therapists wondering how they would catch up, how they should be providing care without that assessment, and how they should safely restart this essential test by respecting public health measures. With mixed messages from the provincial health authorities and professional associations, respiratory therapists spent a lot of time researching spirometry and COVID-19 and cleaning procedures, working to find ways to restart spirometry.

During the disorienting phase, the primary care therapists’ roles were further disrupted by reduced referrals, leaving the therapists wondering what to do, or how to increase referrals. The therapists thought that a main reason for referrals dropping off was due to physicians’ limited understanding of the role of occupational, physical and respiratory therapy. Since the therapists were no longer visible to the team in a physically co-located way, they had fewer opportunities to educate team members about the breadth of their role and the contributions they could make to patient care during the pandemic. The therapists perceived that physicians have limited views of the professions’ roles based on their most common (or most valued) role for the physicians, e.g. home visits for occupational therapy, musculoskeletal care for physical therapy, and spirometry for respiratory therapy. Emma said:

I feel like the understanding of occupational therapy, it’s misunderstood at the best of times. That’s how it feels in community, and I just think that, yeah, there’s so much more that we could be doing to support people living in the community and to use our skills to keep people out of hospital. I just feel like that’s not happening, yeah. Lack of referrals, too many people don’t know we’re at work.

To counter the disorientation from not being able to see patients in-person, the therapists framed this time as an opportunity for “catching up” as a clinician might do at other times when there is an interruption in patient care – for example, during a snowstorm when patients are unable to get to their appointments. The therapists caught up on paperwork, engaged in professional development activities, and updated and created handouts that they would be able to use when things “got back to normal”.

In addition to lost activities, disorientation was furthered by therapists being asked to engage in new pandemic-related activities that, while being within their scope, were not typical for these therapists in primary care. Most were asked to support COVID-19 screening or testing in the first few weeks of the pandemic, requiring them to learn new skills. Complicating this was the lack of consistent information to support them, as Ashley described:

We were asked, um, encouraged to help with screening of people as they walked into our clinical setting. Um, I share my clinical setting with other primary care providers and, um, they needed somebody at the door to screen the clients coming in and this happened very quickly and abruptly and unexpectedly. Um, when we initially starting doing this, it was very unclear as to exactly what we were supposed to be doing, uh, what we were supposed to be asking people, what the protocol was in terms of ourselves, our own, uh, protective equipment as well as what we should be expecting people to do walking in the door. Um, so this was kinda thrust upon us and, um, expect- expected that we- that we do this, myself as well as people on my team. Um, from there, things were really different and changing on a regular basis.

Ainsi explains:

I felt- felt a bit down with anxiety, I guess, we're trying to get things- i- it’s more because of the unknown than anything else. I didn't know what was happening, what was happening with the patients, um, you know, what did it mean for us to have the COVID in Manitoba and, you know, the whole spread thing wasn’t spreading just by travelers. So anyway, that was the start of the COVID for me.

To regain their orientation in this new situation, therapists spent a lot of time trying to learn everything they could about COVID-19. They sought to learn about, and update their knowledge regularly on precautions, including masking and cleaning protocols, so that they could make decisions about care provision. Eva said in an early journal entry,

And we've spent a lot of time sifting through all the information that's come through. Um, being only one of two physiotherapists, um, at our site and not very many, I guess, even in the city, um, we found that we had to sift through the information that was coming from our managers or the medical lead at the site that we're working at and then figure out how that fits for our profession, making sure that we're still following, um, what's appropriate for our profession and what, um, sort of our college requirements are, um, to give good care to our patients.

The combination of trying to follow all the public health orders, and new policies and procedures in addition to trying to shift to new service delivery platforms meant that tasks that were previously routine now needed to be relearned or re-visited in terms of processes. To do so, the therapists needed to consider privacy laws, and their professional colleges’ regulations for tasks such as gaining consent for treatment, to create new service delivery methods. Eva explains:

We spent a lot of time figuring out or, I spent a lot of time figuring out, um, what our college requirements were as far as, uh, making sure we identified the patient, uh, what informed consent was and sort of what were their other, um, choices for treatment so that we knew that they were making an informed decision when they decided to do a phone consultation as opposed to face-to-face, um, and really understanding what were the limitations of those appointments. Um, there was a bit of a learning curve trynna figure out how do we get, um, exercise sheets or information to the patient, um, was it okay to do it via email or not, um, as far as, ugh, PHIA (Personal Health Information Act) on things like that.

Finally, in this phase, the therapists also were disoriented due to disruption of personal routines and roles. Everyone needed to adjust to working at home at least part-time, developing new family routines due to disrupted dependent care, setting up their home physical environment to support more family members being at home attending school and work online) and finding new ways to create boundaries between their home and work life. This in combination with continual policy changes made it difficult for therapists to have energy for adaptation:

I find, uh, professionally through conversation with colleagues, co-workers, um, capacity of the general therapist is- is lower at this time. Not capacity in the sense of competence, capacity in the sense of energy, motivation, um, and capability to take on more. Just everything’s, you know, being re-created, adjusted and like I said modified. (Maria)

### Phase 2: Coping and waiting

In this phase, the therapists started to develop some stability with respect to their environment and with day to day personal and work routines, work policies, procedures, and expectations. Stability provided an opportunity to adjust to the situation and expectations via trial-and-error with new ways of delivering care. However, there was still a sense that they were biding time, waiting for things to “return to normal”. In this comment, Ashley illustrates how therapists quickly developed routines that felt normal to them, even though they were very different from the routines they had only 3 months earlier prior to the pandemic.

I again have nothing really new to report. Uh, my day was a usual day. Um, currently on Thursdays I'm working, in the morning, at the office and seeing patients, etc. And then the afternoon, I am using that time, uh, to work from home, to assist with my childcare needs and using that time to do phone visits and, uh, book my, uh, clients’ appointments and do paperwork and administrative duties. Um, and I'm doing that actually on Fridays as well. So, continuing to spend the majority of the week in the office but sometime at home, um, again, mostly primarily to co- uh, accommodate my- my childcare needs.

Periods of relative stability allowed the therapists to develop skills in delivering patient care within the public health care restrictions. Patient needs that arose or were exacerbated during the pandemic drove therapists to use, learn about, and develop their own best practices for pandemic rehabilitation care. This included pandemic-related mental health needs and repetitive strain injury from working from home with poor ergonomics. There were also some populations that the therapists realized would benefit from continuing their previous interventions but without in-person visits, such as chronic pain psychoeducational groups. The therapists practiced and become more proficient other service delivery methods like telephone and online.

The most striking example was in developing skills for providing care by telephone. The next two comments exemplify the progress Heidi made in using the telephone as a therapeutic platform over the course of a month. Referring to March 2020, she says:

I found those first few phone visits incredibly challenging. I couldn’t read the patient, because I couldn’t see them, and I couldn’t read their body language. Often, a patient will tell me “I’m not short of breath”, but when they walk from the waiting room into my office, you can visibly see the shortness of breath. It would be like doing an exam of a patient with a blindfold on, and I never imagined I would do an exam of a patient with a blindfold on, never mind over the phone. So, here I am trying to do respiratory care through a phone call.

Three weeks later she reports,

Today was probably my busiest day on the phone. I spoke with 15 patients today over the phone. Three or four of those patients were new patients to me that I had never assessed before. All had diagnoses on their charts. Three of the new patients were asthmatics who had uncontrolled asthma. Today, being the start the of spring, there is a lot of snow mold and a lot of allergens in the air, and it is often a time when asthmatics do start to lose control if they don’t initiate their asthma action plans. Again, it was a very, very busy day. I would never seen 15 patients in a clinic in a day, so speaking to 15 patients over the phone is a lot, but troubleshooting and going through the asthma guidelines, working with people and their asthma action plans, moving them into their yellow zone, or changing their medications definitely is still something I can do over the phone. I wouldn’t say it’s ideal, I wouldn’t say moving into the future that the respiratory visits should go virtual or phone-oriented, but what they do allow us to do is to touch base with the patients and make the changes necessary.

As the therapists got more comfortable with the telephone, they found it to be a much more useful tool than they had anticipated. They developed a specific skill set related to phone care: learning about what types of care delivery they could and could not do well with this platform. There was more variability with video-based care because it depended on the therapist and patient having access to the needed equipment. However, for those with the equipment, the learning trajectory was similar to that for the phone.

While *Coping and Waiting*, many therapists did activities related to COVID-19 that had not typically been within their role. This included teaching patients how to use virtual platforms, and how to maintain COVID-19 precautions. They also provided more generalized support to patients, including making sure patients’ basic needs were being met, providing basic mental health support, and connecting people to services as needed. While many of the therapists started these different activities, it was initiated in different ways. Several occupational therapists were assigned the role of making outreach calls by their team members. Physical therapists and respiratory therapists started checking in on general wellness with their current caseload, because they had more unstructured time during their visits than would be typical of an in-person visit. Jenny said,

You know the calls aren’t as long as an hour-long spirometry, and so invariably inevitably you get to speaking about generally how they’re doing. And I’m not saying that I’ve never asked people that in the past, but it’s always been so focused on the task at hand, I guess you could say, but I’ve been able to help people a lot more with, other things that they’re having. I’ll give you an example you know, like ‘I have been having caregiver burnout, or financial strain’ and I get to connect them with resources. Or, I can just listen to them and have time to listen to what’s going on and their frustrations and things like that, which can be a good or a bad depending on which way you look at it. It’s good for them but, after awhile, it’s, it’s an extra emotional burden for the provider but uh. So I have more time to just see how they’re doing, like as a whole, rather than just the respiratory piece. Now, there’s a lot I can’t do about that, but I can get them connected with resources um and they might be more open to accepting those resources. I find that people, it’s uh, it’s been an opportunity to get people connected to resources that they might have been reluctant to receive in the past.

While physical and respiratory therapists felt that it was important to meet these mental health and resource navigation needs, they had different levels of confidence in doing so. Heidi explained,

Most of my phone calls, I feel like I am a counsellor, which I am not, and I feel like I am trying to counsel patients through anxiety and fear. They get my deepest sympathy, because when they started talking, I feel what they are saying, but I’m not a trained counsellor. For me, it is very hard for me to talk people through and walk people through and, again, on the end of a phone where I can’t see their face and I can’t see their reactions. Another thing I never thought I would become [as a RT] was a counsellor, but there is definitely some counselling going on with patients on the phone, and then constantly trying to keep physicians updated as to how their patients are doing.

Extra unstructured time in appointments, previously used for assessment, allowed both physical therapists and respiratory therapists to develop skills in interviewing and information gathering in a more holistic way.

Some therapists were able to broaden their role in additional ways. For example, in Manitoba, some occupational therapists worked exclusively with people with specific chronic conditions prior to the pandemic. However, referral rates were decreasing for chronic condition care. They recognized that there was a broader mental health need in the population because of the pandemic. To address this gap, they started advertising their skills in mental health support to referring physicians.

I may not be able to do home visits, but, look, people are struggling. People aren’t coping. It’s a big change. Refer them, even though it’s not for my designated title role that is a focus. They don’t need you know, a… diagnosis, necessarily. Just send them my way. I want to connect them, kind of like a service navigator or coping assister. (Maria)

As a result, therapists took on roles that were in their professional scope of practice in relation to changing patient needs.

### Phase 3: Adapting - regaining agency as a professional

Not all the therapists reached the ‘a*dapting’* phase during our period of data collection, and some shifted in and out of this phase as their personal, meso, and macro contexts changed over the course of the year of data collection. This phase is marked by therapists having a sense of control and autonomy over the services they offer as well as how they would offer them, and a recognition that these practice changes would continue for some time. In this phase, they had enough knowledge about COVID-19 policies and procedures, types of service delivery platforms and related policies and procedures, and fewer external expectations, all of which allowed them to return to feeling confident for profession-specific decision-making and planning. Further, at this stage, the therapists were capitalizing on the forced innovation that occurred due to the pandemic to improve quality of care in some areas.

Adapting practice to improve quality of care was profession specific. Physical therapists found that being forced to be “hands-off” allowed them to focus more on education and coaching, which are both are activities strongly supported by research. Pre-pandemic, the physical therapists spent less time on these components, because time was taken by hands on assessment (and for some therapists, intervention). Julie talks about how her practice has shifted:

I would say this shift from manual therapy to education. Yeah, I would say previously I would still - like exercise has always been important - but I think previously I would rely on - on my hands a lot more and putting a lot more - what's the word that I'm looking for, like putting a lot more weight on manual therapy and treatment and thinking that we need to release these muscles and I need to move this joint and I need to increase movement here and- and really focusing on that part of it. Whereas now shifting to virtual, the shift has been education and trying to change the way people move during their day and what positions aren't ideal for them. And it's more about kind of giving them the power and the knowledge to be able to make those changes rather then and, I’ll say kind of me as a guide rather than me the one doing the work and them receiving it. So really kind of making the patient a lot more active in their own, in their own rehab. I would say that's been the biggest change. . .

Respiratory therapists found that they were able to implement best practices for chronic care management now that they had confidence in  delivering care in alternate ways. One repeated example of this was smoking cessation. Tammy and Ainsi found that they could provide regular support to those interested in ceasing smoking by telephone, at intervals recommended with best practice guidelines, something previously not possible due the demand for spirometry assessment.

Some therapists developed new programs using online platforms to meet new community health needs, while others worked to shift existing programs from in-person to online. For example, some occupational therapists saw an opportunity to support pandemic mental health and initiated the development of a virtual self-management support and education group for developing strategies to cope with the pandemic. Respiratory therapists supported the development and implementation of new programs, or the expansion of existing programs for addressing COVID-19 needs.

In this phase, the therapists also applied learning about different service delivery platforms to develop their own best practices, balancing efficiency, pandemic safety and patient need. For example, they learned that the telephone was an excellent triage and follow-up tool. Many created a new routine of completing a short initial interview by telephone to determine the best service delivery option (online or in-person), develop an understanding of the presenting problem, and help the patient prepare for the visit. This allowed for better use of their time in virtual and in-person visits. In-person follow-ups pre-pandemic had high “no-show” rates, but therapists found that a telephone call was often adequate to determine if the patient was doing well or needed more intervention. These activity changes were something they planned to continue even when there were no restrictions on in-person service delivery.

Once in *Adapting*, therapists found more success in advocating to physicians and other referrers regarding the broad scope of their role and how they could help with chronic disease prevention and care during this time. Eva shared,

I think when we offer to help with some of the, um, chronic disease management, I do feel like there was sort of a ‘Oh, you can help with that?!’ and ‘What can you do?’ and I think we were able to sort of, offer what we could, and I think they started to use us a little bit more in that realm. Umm, but I think for the most part, the primary care providers think we do musculoskeletal treatment, and so they keep sending the musculoskeletal referrals. And I think they, seem to feel just as confident that we can help now as we could before, because they’re certainly still sending everything. Um, so, yeah, I think that chronic disease part may have changed a little bit during this time.

In this phase, the therapists were able to look beyond their own personal practice and start collaborating with other professionals and sites which was facilitated with the use of virtual platforms. Maria said,

We never worked into intermingling with or interchangeably with other my health teams and other primary care types. And now we're co-running to combine larger groups together and have them run more frequently versus simultaneously different sites. So that's worked really well in the sense of collaboration and, uh, learning and exposure to other teams, processes and roles and just learning about them in their teams. Uhm, and clients are …coming together for themselves like they're not they're not there to mingle with people in their community area. Uhm, yeah, that's that expansion is nice. It does complicate things in different ways, but it's nice overall.

### Snakes and ladders

Keeping with the metaphor of the game, snakes and ladders were factors beyond the individual therapist’s control that could hinder or help role adaptation, respectively. We summarize these in Table 1, at both meso and macro levels.

**Table 1:** Meso and Macro Snakes and Ladders

Factor	Snake or Ladder?	Details
*Meso*		
Team connection and contact	Ladder if frequent/consistent virtual means of contact maintained or initiated	• Therapists whose teams were physically co-located prior to the pandemic described feeling connected to their teams, and vice versa, as they entered the pandemic. • Regardless of pre-pandemic connectedness, therapists’ sense of connection with their team was influenced by the development, or lack thereof, of technology-mediated forms of contact. • Both informal and formal means of virtual contact helped with adaptation, from teams’ social What’s App chat groups, to regularly scheduled formal team meetings. • Exception: certain forms of informal/spontaneous in-person interactions that had previously helped with communication of role clarity and prompted referrals from the team were difficult to replicate with technology.
Immediate supervisor support	Either, dependent on level of support of supervisor	• Supervisors were perceived to remain consistent in their pre-pandemic management style. • Supervisory support was important during the pandemic as policies and procedures changed continually, having a direct impact on the therapists everyday work in predictable and unpredictable ways. • Supportive supervisors ensured that there was transparent two-way communication between themselves and the team and provided reassurance and support. • Early in the first year of the pandemic, therapists appreciated immediate supervisors who were directive, but who transitioned into a more supportive role as time brought more stability. • Leadership changes and inaccessible immediate supervisors left the therapists feeling alone in navigating day-to-day procedural changes.
Access to resources	Either, depending on access or lack thereof	• PPE: a particular concern in the first months of the pandemic, where pathogen transmission method was unclear. At this point, PPE was scarce in primary care, and some were asked to reuse PPE, creating fear and anxiety. • Computer hardware and software: the speed of access to computers, and software needed to meet privacy standards, influenced adaptation. • Clerical support: new clerical demands included setting up virtual appointments, ensure patients knew how to use the technology, mailing out information to patients, screening patients for in-person appointments, room cleaning after in-person appointments. Mainly a snake for therapists because there was no or limited clerical support. Therapists did these tasks themselves, reducing time for patient care.
Within-profession colleagues	Ladder	• Therapists who sought support from other members of their profession working in primary care found that helped adaptation.
Support for virtual care	Either, dependent on setting/organization	• Most organizations pre-pandemic had policies and procedures that limited the use of virtual care due to concerns of privacy. How quickly these polices changed, and the therapists degree of access to the needed computer hardware and software, affected adaptation.
*Macro*		
Organizational Leadership and Meso/Macro Coordination	Snake	• Almost all therapists described the leadership above their immediate supervisor to be a source of frustration that impeded role adaptation. They reported a lack of communication and coordination from upper-level administration, and between macro and meso structures. This resulted in uncoordinated information from varied sources that they had to work to interpret. • Therapists they felt higher levels of management had little understanding of their work and work context, and did not value their contributions and sacrifices made, while other professions received kudos and financial bonuses. • There were a lack of feedback channels with the higher levels, and thus little opportunity for input into planning or decision-making. • In Manitoba, this dissatisfaction intensified over time. After a very limited first wave during spring 2020 and a long period of few cases in summer, the Manitoban therapists were frustrated at the persistence of earlier patterns of poor communication and disorganization. They interpreted this as minimal planning or preparation for the second wave, despite signs from other places showing its eventuality. This disorganization was especially evident in second wave redeployment.
Redeployment	Either, dependent on personal and organizational factors, but mostly a snake.	• Redeployment was the most stressful factor influencing role adaptation. • Most therapists were redeployed to activities outside of usual scope, and many outside their usual primary care organization. • Redeployment was an opportunity to do something concrete and that felt helpful during early weeks, when the therapists were disoriented and struggling with uncertain and daily changes. • However, therapists were overqualified for some redeployments, which when sustained, led them to feel that their expertise was not understood or respected, and created a lack of meaningfulness in their work. • This work took away from work that would have a bigger impact on the pandemic response. • In Manitoba in particular, a major snake was the lack of communication and coordination regarding redeployment. Some were told they were going to be redeployed for the second wave of the pandemic, but not when or where, which left them in limbo for weeks regarding taking on new patients, scheduling follow-up visits, or prematurely wrapping up patient care in preparation for redeployment. • Redeployment was positive for some therapists. Positive aspects included creating in-person connections with known and new colleagues, learning more about other programs in primary and public care, and creating new opportunities for skill development.
Regulatory bodies	Snake early on, then ladder for some when they developed and released virtual practice guidelines	• Initially, pre-pandemic regulatory body guidelines for topics such as consent processes prevented virtual care, slowing adaptation. • When regulatory bodies did release virtual care guidelines, they were welcomed for their guidance.

### Personal influences

Finally, we draw attention to personal influences on how each therapist navigated the pandemic’s disruption in both their personal and professional lives. Individual circumstances and personal characteristics shaped role adaptation, in ways that helped or hindered. For example, balancing work and home life demands was made more challenging for several therapists who were working from home while their school-aged children attended virtual school. This could both split their attention, and put extra demands on resources, such as internet capacity. As well, different therapists navigated life events such as a spouse’s layoff, a parent’s death, and a marriage. But some of the therapists had been looking for new professional opportunities just as the pandemic started. Through redeployment, they found opportunities for career advancement and development in line with their personal goals. In particular, redeployment into pandemic-related team leaders and education roles helped two therapists embrace the changes and allowed them to demonstrate and develop different skills, such as in evaluation and leadership.

## Discussion

The pandemic provided an opportunity to learn about role adaptation of primary care therapists, which can aid understanding of how to strengthen team-based primary care as well as primary care therapist roles in disasters. Strengthening team-based primary care has been identified as a priority by researchers who have looked back at the COVID-19 pandemic response [12, 23]. Priorities for strengthening primary care include improving health promotion and prevention services in the primary care system [23], and better defining the role of primary care in pandemics, including planning how to be able to continue with day-to-day care for people during times of change [12]. Our study provides evidence that primary care therapists can support these goals, in supportive conditions.

As theorists predict, our findings highlight that role adaptation is clearly possible, but is relational rather than solely under the control of the person [28] and can be a demanding and emotionally difficult experience [29]. The former is illustrated in the many different snakes (barriers) and ladders (facilitators) that shaped regaining a clearer and more comfortable sense of role over time. While a difficult experience for many primary care therapists, the pandemic allowed for practice shifts that better aligned roles with best practices in primary care (such as strengthening self management education and support). To make these role changes, therapists needed both support and autonomy, which not all experienced in meaningful ways. In this discussion, we situate our findings in existing literature and think through how we might support and shorten the more difficult phases of role adaptation during disasters like pandemics. We also hypothesize what these findings teach us about supporting role adaptation to maximize team-based care in primary care for day-to-day care delivery and perturbations.

### Implications for disaster preparation

Supporting therapists to adapt in disasters is important because of anticipated future pandemics and environmental disasters due to climate change [12]. In early weeks of the pandemic, therapists held in common a sense of disorientation, where they were no longer clear about their role and lacked access to important supports for role transition. Therapists were aware of the unmet needs of some of the clients they served, yet unable to offer services to them consistently for reasons out of their control such as redeployment or lack of resources to offer virtual care. However, over time many therapists in our study were able to align their services with client needs in the pandemic as well as chronic disease guidelines, even without in-person appointments.

Other research conducted with primary care teams also describes very important roles for therapists in this type of disaster situation. For example, Ashcroft and colleagues [40] found that there was an increased population health need for mental health supports, an area of practice that occupational therapists can deliver. Additionally, virtual care was found to be effective for many of the services that can be offered by primary care therapists, such as health prevention and promotion, chronic disease management, and wellness checks [40]. This was confirmed by Reddy and colleagues [41], who found that early in the pandemic, American primary care team members were instrumental in supporting veterans’ health; while physician visits decreased, allied health team members increased the number of visits they provided. Activities important to population health in a disaster situation fall under the scope of primary care therapists [23, 42]; this needs to be more widely understood and integrated into disaster planning to support community health in disaster situations to shorten the disorientation component of adaptation in a disaster.

The concept of adaptation phases in a disaster has been written about previously, and the similarity of findings points to future directions. It is critical to include all interprofessional team members in disaster planning to enhance response [12]. In relation to organizational recovery after Hurricane Katrina, Doerfel and colleagues [43] described four phases: personal emergency, professional emergency, transitional, rebuilding. While that disaster was different in nature than the pandemic, some findings of their study parallel our own. First, Doerfel and colleagues describe how people needed to tend to their own personal emergency situation, as our participants did during the *Disorientation* phase [43]. Second, Doerfel and colleagues also describe a transitional phase, in which the organization assesses the situation. They found that one-way communication dominated at this time, both incoming and outgoing. Incoming communication provided information and support (e.g., communication about new policies) and outgoing communication sent information to other organizations or clients (e.g. telling clients about cancelled appointments). Doerfel and colleagues suggest involving employees in this transitional phase to start recovery [43]. In our data, we did not see evidence of meaningful involvement of the therapists in recovery discussions and activities, potentially keeping the therapists in stage of disorientation longer than necessary. Similarly, in Australian general practice, problems arose in practices where physician leadership failed to include the range of professionals in decision-making compared to those with more inclusive approaches [44].

Doerfel and colleagues also highlight how connecting beyond one’s own micro and meso environment can shorten the transitional phase and support movement into the recovery phase [43]. In contrast, it may be that the pandemic created the unique circumstance of people not being able to connect easily for a prolonged time, thus lengthening the transitional phase, keeping the therapists in the *Coping and Waiting* state rather than allowing them to move more quickly into *Adaptation*. Overall, Doerfel and colleagues [43] highlight the importance of having policy and procedures in place that can be enacted in a disaster to shorten the earlier phases of a disorientation, and support micro, meso and macro levels to connect quickly and communicate reciprocally to move into *Adaptation* as quickly as possible. This illustrates that if interprofessional roles in primary care settings are better understood, teams can plan ahead for different scenarios, and shorten their *Coping and Waiting* state and move into action more quickly.

Our study validates other research findings on the barriers to adaptation of professionals during the pandemic. One of these was the need for safety-oriented supports early in the pandemic [44]. Health care systems need to be more prepared for disasters in relation to PPE supply and demand plan, with a need for local capacity to produce PPE when required [45]. Patel and colleagues [45] argue that primary care facilities can be better designed to support the prevention of infectious disease spread and still support collaborative environments, reducing the strain on therapists we observed from needing to have a continual high level of diligence to infectious pathogens while carrying out their daily work. Barriers to the transition to virtual care are also discussed in the literature. Greenhalgh and colleagues [46] wrote about the kinds of supports primary care would need to rapidly introduce virtual visits, including training on the mediums, guidance for both therapists and patients on how to make the best of the technology, and in-person technical support. In both our study, and Donnelly and colleagues’ [42] survey of interprofessional primary care team members in Ontario, these virtual care supports were limited to non-existent, slowing adaptation.

Some of our findings on organizational support resonate with other studies, while some are unique. Similar to our study, Donnelly and colleagues [42] found that the pandemic had a variable impact on team collaboration, with 48% percent of survey respondents reporting that team collaboration increased during the pandemic, and 33% reporting less collaboration. Our results provide some insight into the reasons for this, with the intentional use of electronic communication methods and strong team leadership being strong facilitators – a finding echoed in a study of six Australian general practices weathering the first 9 months of the pandemic [44]. A unique finding on our study was the importance of within-profession support. Primary care therapists are often the sole person of their profession on their team, and typically do not have a manager or leader who shares their profession. This left some therapists with no support for professional specific issues early in the pandemic. Also unique was that therapists felt that professional associations and regulatory bodies were not fast enough in supporting their membership, pointing to the need for these organizations to also undergo disaster preparation planning.

The biggest ongoing challenge cited by therapists in our study was communication and collaboration within and between meso and macro levels, with problems persisting through the entire year. An example of the impact of poor communication was that primary care therapists felt that they were redeployed to activities that were not in their typical scope of practice or were activities that could be done by non-licensed health professionals, resulting in missed opportunities for health professionals to support existing clients or broader population health needs. In Manitoba, this lack of communication continued 9 months into the pandemic, indicating that this type of bidirectional communication was not prioritized well past the initial early pandemic response. Work by Sangal and colleagues [47] on communication between front line providers and managers during the COVID-19 pandemic suggested that consistent and frequent communication from managers was an important support for front line providers during the pandemic, and mitigated feelings of stress and burnout. In addition, previous research has indicated that two-way informal communication is key to developing and sustaining collaboration, and that co-location facilitates this type of communication by promoting frequent face-to-face communication [48, 49]. In the pandemic environment, with team members forced to work in physically divided spaces or remotely, this important avenue for promoting team cohesion and communication was lost. It was vitally important to find alternate ways to maintain this type of communication [50]. If there had been more bidirectional communication between front line providers and administration during the pandemic, the primary care therapists may have been able to better communicate how their scope of practice could support the pandemic response [47].

### Implications for role development in daily team-based primary care

The findings of our study can also inform the continued development of therapist roles in primary care settings. Our study confirmed other findings that physicians and other primary care team members still have much to learn about the role of primary care therapists [5, 7, 10], since losing the ability to provide informal education on their resulted in a drop in referrals for services. Ensuring that teams understand the full scope of primary care therapy roles can allow primary care to better support the growing population living with chronic diseases. Research to date on primary care therapy roles and scope has only identified what therapists are currently doing, rather than exploring the broader potential of their contributions in primary care [4, 9]. In our study, therapists took a larger role in chronic disease and mental health care than they had previously done in these settings, thus aligning patient care better to chronic disease guidelines. Thus, the turmoil of the pandemic revealed promising practices for therapists to contribute to population health in primary care settings following the pandemic. Following the pandemic, it would be regretful if primary care therapists simply returned to their previous narrower roles (e.g. spirometry assessment for respiratory therapists) rather than maintaining expanded roles that implemented a broader range of chronic disease best practices. Dialogue about important gains made within patient care by primary care teams should occur, and teams should be resourced to build on successes.

The most significant application for our findings in the current context is the primary care physician shortage. To address this shortage, it is necessary to maximize the use of other health professionals in primary care [22]. The literature cites a few successful initiatives where primary care therapists supported efficient and effective primary care delivery. For example, physical therapists can be a first point of contact for musculoskeletal complaints [25], and respiratory therapists can provide chronic respiratory condition care. However, these examples are exceptions rather than standard practice in primary care settings. If therapists are supported to adapt to a fuller scope of practice or be involved in determining how to adapt to fill a particular need within a primary care practice, therapists can provide more support for day-to-day primary care services [7]. Meaningful engagement of primary care therapists in care services planning needs to occur for therapists to have work satisfaction and practice to their optimal abilities [47, 51]. This type of planning will also need to consider necessary resources for primary care therapists, so they are not faced with barriers in providing services, as they were during the pandemic with a shortage of technology and support staff to adapt their work roles. As primary care transitions to more team-based care, the roles of the primary care therapists as well as resources needed to fulfil these roles efficiently need to be factored into health care planning.

Finally, understanding role adaptation is important because health care is never a static entity; the needs of populations and the context in which care is being delivered is constantly shifting. When team members understand each other’s roles, the team can adapt to periodic shifts in population care needs, such as flu season, or to shifts in their own team context, like when a team member with particular expertise leaves their position.

Considering disaster planning and supporting role adaptation together, including primary care therapists in pandemic planning could identify ways to transform primary care practice that supports current team-based practice as well as support improved flexibility for disaster responses. For example, Donnelly and colleagues [42] suggest that health provider direct access, rather than a physician referral model, could combat the reduction in referrals in the early phase of a disaster, when physicians are busy with early response demands and may have a limited understanding of roles of the other team members during this disaster phase. Exploring this option could reveal how this model could be used in day to day primary care to improve system efficiencies [25].

### Influences of individual experience in role adaptation

Our study suggests factors to focus on to support role adaptation for primary care therapists. However, organizations need to also keep in mind that personal factors will also always facilitate or impede personal adaptation. For example, in our study, some therapists were ready for a new professional opportunity at the beginning of the pandemic, including the challenges such moves bring. This readiness helped them to move into adaptation faster than some of the other participants, for example, those new to the primary care role, or those who had specific personal challenges due to the pandemic such as a spouse with job loss or small children requiring childcare. To support clinicians, preparedness needs to have mechanisms to consider the range of needs of clinicians, an issue that has been previously flagged in Canada based on the first SARS outbreak in 2003 [52].

### Strengths and limitations

Our choice of a longitudinal qualitative design, and use of iterations of analysis, are strengths. We were able to follow the same therapists over time, developing a rich understanding of each of their situations, though we experienced some attrition. We did not collect information on gender, which may be a limitation since caregiving, both professional, and personal, is gendered. The iterations of analysis and data collection allowed us to ensure interview guides addressed emerging insights in a situation about which little was previously known. And the use of multiple data sources and analysts are two triangulation methods that enhance the quality of qualitative research [53]. This depth comes at the trade off of breadth; we limited our sample to three professions working in primary care settings in two Canadian provinces. Like all qualitative studies, transferability to other settings or professions is left to the reader to decide.

## Conclusion

Primary care occupational, physical and respiratory therapists can adapt services to better meet population needs and provide crucial services to support population health both in disasters, and in day-to-day primary care delivery. The ability and speed at which these primary care therapists can make adaptations to support their team and the needs of the population being served depends on team, organizational, and policy-level supports, as well as personal factors. Future research should focus on best practices and implementation for integrating primary care teams into primary care planning, including disaster planning. As policy makers take stock of the lessons of this pandemic, and improve pandemic preparedness planning, we and others encourage better integration of primary care and the varied professions within that sector [40, 42], to capitalize on what many have agreed primary care is best-positioned to do: meet the needs of the vast majority of the population.

### Supplementary Information

**Additional file 1: Appendix. **Semi-Structured Interview Guides.

## Data Availability

The datasets used and/or analysed during the current study are available at an aggregated level to protect participant confidentiality from the corresponding author on reasonable request.

## Abbreviations

PPE: Personal protective equipment
