# Supplementary material for: Primary care occupational, physical, and respiratory therapy role adaptation in the first year of the COVID-19 pandemic
Source: BMC Prim Care. 2024 Jan 2;25:3. doi: 10.1186/s12875-023-02247-7 (PMC10759467; doi:10.1186/s12875-023-02247-7)
Supplement: Supplementary file 1 — Additional file 1: Appendix. Semi-Structured Interview Guides. [file 12875_2023_2247_MOESM1_ESM.docx]

***Appendix: Semi-Structured Interview Guides***

***Interview 1***

**Micro**

1. At the time of your last diaries in (date), you were (SUMMARIZE). Please catch me up on what’s happened since
2. How similar is your current work with your pre-COVID responsibilities?
3. Imagine you had full freedom to decide what you do in your workday, during this pandemic. What would you be doing?
4. Did you see a difference in the patient population that you see now, compared to before the pandemic
5. How do the changes in service delivery impact your patients?
6. Do you think there are some patients that were unable to access your service?
7. Since the beginning of the pandemic, what have you done that has been the most helpful for your patients?

**Meso (teams)**

1. When you talk about your team, who do you consider as being part of this?
2. Over the course of the pandemic, how has your work within your primary care team changed?
3. Do you feel supported by your primary care team during the pandemic?
4. If redeployed:
5. In your diary entries, you mentioned being redeployed. Tell me about that experience.
6. 5 -What do you think are the big challenges in healthcare needs in your community right now?

**Macro**

1. Think about your provincial government. What things have they done that have been most helpful in response to the pandemic? Do you think there is something that the provincial government could do to provide some support to you, or your primary care team, moving forward.
2. Think about your profession’s regulatory body and provincial association. What things have they done that have been most helpful in response to the pandemic? What supports do you think would help, moving forward, from your regulatory body?
3. In your opinion, how well has your profession been integrated into the response to COVID19?

***One-year follow-up interview:***

- Are you still working in the same workplace as when we spoke to you last? If not, where are you working now?
- Review what the participant shared at post- diary interview and ask what is new since that interview.
- I would like to have you reflect back on the entire year. What has been the most significant change to your work as an (OT/PT/RT) since the start of the pandemic?
- Thinking back over the last year, what has been the biggest changes to your scope of practice/role?
- Looking back over the past year. What are the most significant changes in *how* you do your job?
- Looking back over the past year, what are the most significant changes in who you serve?
- What would you say was your greatest success in adapting to COVID19 in your professional role? What supported you to make this adaptation?
- What was most difficult when adapting to COVID19 in your professional role?
- Looking back, what other supports or resources would have helped you that haven’t mentioned yet?
- What have you learned about yourself during this COVID-19 pandemic in relation to your professional identity and role?
- Is there anything else you want to tell us about how you adapted during the COVID19 pandemic as an (OT/PT/RT) and what supports and resources did support you, or would support you in the coming months?
